# From endocrine to immune: therapeutic potential of brown adipose tissue in remodelling the cardiovascular immune microenvironment

**DOI:** 10.3389/fimmu.2026.1895204

**Published:** 2026-09-23

**Authors:** Yuanpeng Liao, Senping Xu, Zhixiang Liao, Jiawei Guo

**Affiliations:** 1 Department of Vascular and Endovascular Surgery, The First Affiliated Hospital of Yangtze University, Jingzhou, China; 2 Department of Pharmacology, School of Medicine, Yangtze University, Jingzhou, China

**Keywords:** adipose-targeted therapy, batokines, brown adipose tissue, cardiovascular immunity, immunometabolism, perivascular adipose tissue

## Abstract

Brown adipose tissue (BAT) has transcended its classical role as an energy-dissipating thermogenic organ, emerging as a pivotal endocrine and immunoregulatory hub. This review systematically delineates the novel BAT endocrine mediator-cardiovascular immune microenvironment axis. We elucidate how BAT-derived secretomes, encompassing specific batokines, lipid mediators and extracellular vesicles(EVs), orchestrate profound cross-organ crosstalk to modulate systemic cardiovascular immunity. Pathophysiological stress, including obesity and ageing, precipitates BAT whitening and dysregulates local neuro-immune-metabolic reflex arcs, thereby exacerbating atherosclerosis(AS), hypertension and heart failure. Crucially, the phenotypic transformation of perivascular adipose tissue(PVAT) is intimately linked to localized vascular inflammation and macrophage polarization. Furthermore, we critically evaluate cutting-edge pharmacological interventions targeting key pathways, including FGF21 signaling, BAT-derived exosomes, and specific lipid mediators. These therapeutic modalities span conventional adrenergic activators, advanced nanotechnological delivery systems and cell-free epigenetic therapies. By circumventing the cardiovascular toxicities of systemic sympathetic overactivation, these targeted strategies precisely remodel the adipose microenvironment. Ultimately, harnessing the profound immunometabolic plasticity of BAT unveils a groundbreaking therapeutic paradigm for combatting advanced cardiometabolic diseases.

## Graphical Abstract

**Figure:**
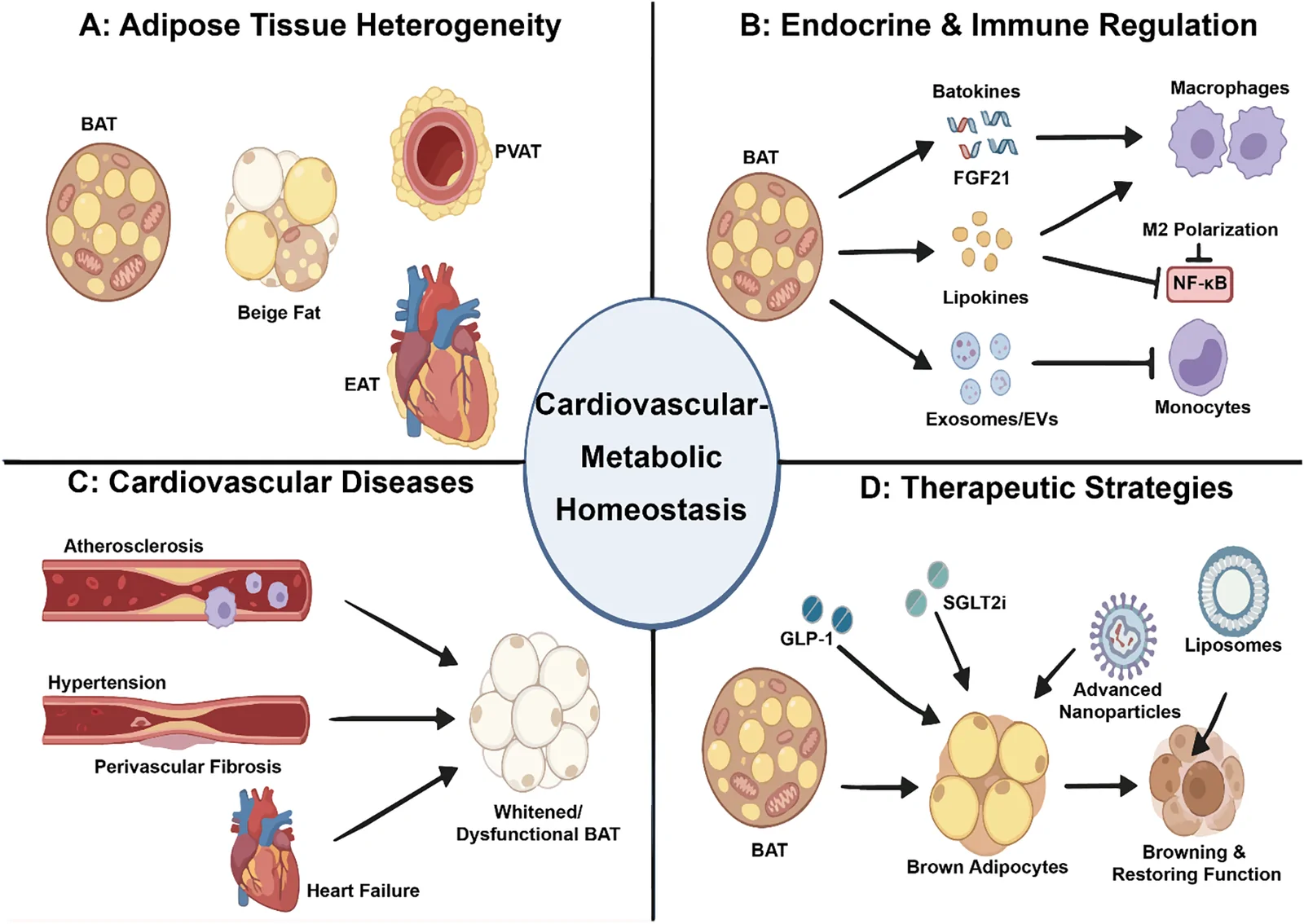
The multifaceted role of BAT in cardiometabolic homeostasis and targeted therapeutics. **(A)** BAT Heterogeneity: Adipose depots exhibit distinct profiles comprising classical BAT, beige fat, PVAT and EAT. **(B)** Endocrine and Immune Regulation: BAT operates as a robust secretory hub releasing lipokines, Evs and batokines including FGF21 and NRG4. Consequently, these secretions modulate immunity by driving macrophage M2 polarization, attenuating NF-κB signaling, and restricting monocyte infiltration. **(C)** Cardiovascular Diseases: Pathologies including atherosclerosis, hypertension, and heart failure precipitate BAT whitening and metabolic dysfunction. Therefore, this transition generates a pro-inflammatory microenvironment exacerbating cardiovascular injury. **(D)** Therapeutic Strategies: Restoring BAT activity and promoting browning constitute promising cardiometabolic interventions. Furthermore, these approaches harness pharmacological agents such as GLP-1 receptor agonists and SGLT2 inhibitors alongside precision delivery systems including nanoparticles and liposomes.

## 1 Introduction

The pathophysiological foundation of cardiovascular disease (CVD) is fundamentally rooted in chronic low-grade inflammation driven by metabolic dysregulation. Beyond merely buffering excess calories, adipose tissue functions as a central metabolic hub and dynamic endocrine platform crucial for maintaining systemic energy homeostasis (1). During caloric excess and metabolic stress, pathological adipose expansion and hypertrophy precipitate local hypoxia and impaired microvascular angiogenesis (2). Consequently, this adverse remodelling triggers extensive immune cell infiltration and a surge in pro-inflammatory cytokines, ultimately disrupting cross-organ communication networks (3). Furthermore, adipocyte dysfunction and aberrant proliferation propel systemic metabolic inflammation (4) and target-organ insulin resistance (5), thereby accelerating obesity-associated chronic pathologies (6). Crucially, adipose dysfunction serves as a primary mechanistic driver in the pathogenesis of AS (7) and exacerbates broader metabolic syndromes, including hepatic steatosis (8). Conversely, systemic vascular disease and endothelial dysfunction can deplete thermogenic fat depots, further aggravating systemic bioenergetic disturbances (9).

The confirmation of active BAT in adult humans via molecular imaging (10) has profoundly shifted perspectives on its therapeutic potential for cardiometabolic complications (11). Traditionally, BAT was regarded solely as a thermogenic organ reliant on uncoupling protein 1 (UCP1) to dissipate energy from fatty acid and glucose oxidation (12). In this classical paradigm, it operated merely as a peripheral effector modulated by central nervous structures like the hypothalamus to mitigate hypothermia (13). Although this robust capacity for uncoupled oxidative phosphorylation positions BAT as an attractive therapeutic target (14), energy dissipation alone remains insufficient to counteract the extensive networks of cardiometabolic diseases (15).

Recent advances have redefined BAT as a highly active endocrine and immunoregulatory organ. Functioning as a vital metabolic reservoir, BAT actively sequesters and degrades circulating lipotoxic metabolites (16), thereby exerting an indispensable extrahepatic compensatory role against systemic lipid overload (17). Consequently, its potent substrate clearance capacity substantially reduces plasma lipid concentrations, mitigating atherosclerotic plaque development (18) and establishing a novel defence mechanism for cardiovascular homeostasis (19). Concurrently, BAT secretes a diverse repertoire of bioactive molecules termed batokines (20). These mediators encompass specific regulatory peptides (21), growth factors (22) and encapsulated signals including EVs and microRNAs (miRNAs) (23). Moreover, batokines modulate systemic metabolism via intricate autocrine, paracrine and endocrine pathways (24), playing a central role in sustaining immune crosstalk between tissue-resident macrophages and peripheral organs (25). Within this functional microenvironment, UCP1-independent endocrine mechanisms exhibit significant therapeutic potential (26). Additionally, the structural and functional integrity of local thermogenic core proteins is essential for sustaining the immunological homeostasis of adipose tissue (27). Importantly, macrophages localized within these thermogenic niches are indispensable for the adaptive regulation of systemic energy balance (28).

Regarding physiological stress adaptation, the bidirectional plasticity between energy-storing white adipose tissue(WAT) and thermogenic brown or beige adipocytes constitutes a resilience-adipose axis, linking systemic metabolic health to environmental demands (29). Because human obesity and ageing frequently precipitate a pronounced decline in core BAT thermogenesis (30), the targeted activation of classical BAT or the induction of white adipose browning (31) has garnered considerable attention as an innovative therapeutic strategy (32). This structural remodelling has been shown to ameliorate obesity and insulin resistance (33) while serving as a cutting-edge translational target to attenuate global cardiovascular risk (34). Building upon the recent discovery that the central nervous system utilizes adipose tissue as a relay station for peripheral cardiovascular protection (35), this review introduces the novel concept of the BAT endocrine mediator-cardiovascular immune microenvironment axis. Accordingly, we aim to elucidate that BAT activation not only enhances systemic metabolic buffering but also has the potential to modulate immune cells via the precise release of endocrine mediators, thereby contributing to profound cardiovascular and immunoprotective effects.

## 2 Distinctions between BAT and other adipose tissues

### 2.1 Histological and molecular physiological distinctions between classical brown and white fat

Mammalian adipose organs exhibit profound anatomical and physiological heterogeneity. Based on ultrastructure and function, they are primarily classified into energy-storing WAT and energy-dissipating, thermogenic BAT (36, 37). WAT stores excess metabolic substrates as triglycerides within large, unilocular lipid droplets and contains sparse mitochondria. In contrast, classical BAT is characterized by small, multilocular lipid droplets, an extremely high density of mitochondria and endomembrane components and robust expression of UCP1 to dissipate energy (11, 12, 38). To maintain this ultrastructure, the endoplasmic reticulum membrane protein CLSTN3β localizes precisely to the endoplasmic reticulum-lipid droplet interface, strictly restricting excessive lipid expansion and preserving the defining multilocular morphology of thermogenic fat (39).

Molecularly, BAT specifically overexpresses Alas1, the rate-limiting enzyme in heme synthesis, at levels comparable to cardiac and skeletal muscle, establishing the foundation for its heme-rich composition and distinctive coloration (40). Furthermore, to meet high metabolic demands, BAT features an exceptionally dense microvascular network and sympathetic innervation (6), wherein neurogenin 4 (Nrg4) critically promotes angiogenesis (41). In adult humans, active BAT primarily localizes to the cervical, supraclavicular and perirenal regions (42). Emerging single-nucleus RNA sequencing reveals profound internal heterogeneity. Specifically, a high-Cyp2e1-expressing subpopulation exists in human deep cervical fat, whereas a lipogenic brown adipocyte subpopulation driving thermogenic memory resides peripheral to high-UCP1-expressing cells (43, 44). However, during ageing or prolonged metabolic stress, FSTL1-expressing progenitor cells lose differentiation capacity. Consequently, this leads to BAT avascularization, fibroinflammation and aberrant lipid accumulation, ultimately culminating in morphological whitening and exacerbated systemic metabolic dysfunction (45, 46).

### 2.2 Developmental origins and sequential thermogenic plasticity of beige adipose tissue

Beige adipose tissue, originating from distinct developmental lineages, can form within WAT in response to specific stimuli, demonstrating profound tissue plasticity (47). Morphologically, beige adipose tissue resembles classical BAT by containing multilocular lipid droplets and expressing UCP1; however, its mitochondrial density and absolute thermogenic efficiency are comparatively lower (48). Unlike congenitally developed classical BAT, beige adipocytes primarily reside within subcutaneous white adipose tissue (sWAT) or inguinal white adipose tissue (iWAT), undergoing browning upon cold exposure or sympathetic activation (49).

During cold stress, mammals mount a highly coordinated, sequential thermogenic response. Initially, classical BAT acts as the first line of defense by rapidly activating thermogenesis. Subsequently, under sustained cold exposure, iWAT degrades the transcriptional corepressor NCOR1 via the ubiquitin ligase RNF20, thereby activating PPARγ and gradually driving precursor differentiation into beige adipocytes to maintain secondary thermoregulation (50). This site-specific remodeling potential is strictly constrained by local genetic factors. Notably, the visceral fat-specific expression of the Wilms tumor gene product (51) and GREM2 suppresses the browning program (52). Concurrently, branched-chain keto acids, acetyl-CoA and the deubiquitinating enzyme BAP1 epigenetically blockade core thermogenic gene transcription, thereby preserving pure WAT characteristics (53).

### 2.3 Brown-like properties and adaptive remodelling of PVAT

PVAT constitutes a distinctive fat reservoir that provides physical support while functioning as a highly active endocrine and paracrine organ, participating in the regulation of vascular tone and hemodynamics (54). Under physiological conditions, thoracic aortic PVAT exhibits high cellular and proteomic homology with classical BAT, featuring abundant thermogenic markers including UCP1 and the mitochondrial stress protein GRP75 (55).

Furthermore, the specific knockout of BAT-associated PPARγ induces classical BAT pathology and impedes PVAT development, confirming a close symbiotic relationship between these tissues (56). Additionally, the PVAT secretome is highly enriched in neural cell adhesion molecules and axon guidance proteins, reflecting a denser network of sympathetic nerve projections compared to other adipose depots (57). In response to stressors including vascular endothelial injury, PVAT spontaneously undergoes beige transformation. Through this localized adaptive remodeling, it downregulates tissue inflammation and functions as an anticontractile vasoprotective barrier (58, 59).

### 2.4 Cardiac-specific phenotype of epicardial adipose tissue

Epicardial adipose tissue (EAT) and pericardial fat, which directly contact the myocardium without fascial barriers and share a microcirculatory network, exhibit unique characteristics in regulating cardiovascular homeostasis (60). Embryologically homologous to BAT, EAT exhibits active thermogenic properties from early life. Although classified as visceral WAT, EAT displays a typical brown or beige phenotype characterized by a higher fatty acid metabolism rate than systemic visceral fat and robust expression of proteins including UCP1, thereby providing dual bioenergetic and mechanical protection to the myocardium (61, 62). Furthermore, coronary-associated EAT cells maintain a significantly dedifferentiated state. This biochemically suppressed phenotype corresponds to local hemodynamics and a complex immune microenvironment, highlighting the trans-domain role of cardiac-resident fat in modulating endothelial function (63, 64).

### 2.5 Heterogeneity of adipose tissue: comparative metabolic and immunological profiles of BAT and other fat reservoirs

Adipose tissue metabolic heterogeneity is clearly reflected in the compositional differences of resident immune cell pools. To maintain local homeostasis, both BAT and WAT adipocytes regulate metabolic and immune balance by transferring mitochondria to tissue-resident macrophages (65). Nevertheless, their respective immune microenvironments remain markedly distinct. Specifically, human subcutaneous WAT harbors higher concentrations of natural killer cells, monocytes and macrophages compared to visceral fat (66).

In BAT, the macrophage pool exhibits high tissue specificity. Notably, Rarres2-positive macrophages are highly enriched in BAT but rarely detected in WAT, requiring continuous replenishment from circulating monocytes for maintenance (67, 68). Moreover, tissue-resident macrophages display a phenotype distinct from classical inflammatory monocytes, specializing in the secretion of specific mediators including PDGFcc to regulate energy storage (69). However, under pathological conditions including obesity or ageing, the excessive accumulation of free fatty acids induces adverse remodeling, precipitating massive macrophage infiltration and the formation of crown-like structures (70). Consequently, this excessive macrophage accumulation becomes the primary source of local chronic low-grade inflammation. This not only accelerates the morphological whitening of classical BAT and the loss of thermogenic genes but also drives the PVAT microenvironment to be dominated by infiltrating pro-inflammatory macrophages during hypertensive stress. Ultimately, this initiates a complete loss of vasodilation function and exacerbates cardiovascular pathology (28, 71).

To clearly delineate the profound heterogeneity among these distinct adipose fat pads, we summarize their primary anatomical locations, developmental origins, and distinct mechanisms of action in cardiovascular pathogenesis in **Table 1**.

**Table 1:** Anatomical, developmental, and functional heterogeneity of major adipose tissues in cardiovascular biology.

Adipose tissue type	Anatomical locations	Developmental origins	Main functions & current research focus	Specific roles in CVDs	References
WAT	Subcutaneous, visceral	Derived from Myf5-negative mesenchymal precursors	Energy storage, mechanical buffering, systemic lipid mobilization	Systemic: Ectopic lipid deposition and pro-inflammatory adipokines drive systemic insulin resistance and atherosclerosis	(51, 72)
Cervical/Supraclavicular BAT	Cervical, supraclavicular, axillary	Derived from paraxial mesoderm	Rapid thermogenesis, systemic metabolism and endocrine regulation	Systemic: Releases protective “batokines” to alleviate systemic inflammation, and protect against heart failure	(42, 73)
Perirenal BAT	Around the kidneys	Derived from Myf5-positive and intermediate mesoderm	Thermogenesis, local renal microenvironment regulation	Local: Modulates renal hemodynamics and inflammation, indirectly affecting hypertension	(74)
Beige Adipocytes	Interspersed within subcutaneous/inguinal WAT	Transdifferentiated from mature WAT or derived from Myf5-negative precursors	Adaptive thermogenesis	Systemic: Browning of WAT improves systemic cardiometabolic health against metabolic stress	(47, 49)
PVAT	Surrounding large arteries and microvessels	Thoracic PVAT originates from Myf5-positive lineage	Regulates vascular tone and local vascular biology	Local(Paracrine): Acts as an anti-contractile barrier; browning limits vascular inflammation and prevents atherosclerosis	(54, 75)
EAT	Between myocardium and visceral pericardium	Derived from splanchnic mesoderm.	Myocardial energy supply, mechanical protection and cardiac-resident immunity	Local(Paracrine): Directly modulates endothelial/myocardial function; whitening exacerbates arrhythmias and HFpEF	(60, 61)

BAT, brown adipose tissue; CVDs, cardiovascular diseases; EAT, epicardial adipose tissue; HFpEF, heart failure with preserved ejection fraction; Myf5, myogenic factor 5; PVAT, perivascular adipose tissue; WAT, white adipose tissue.

## 3 The local homeostatic microenvironment of BAT: A network of metabolic, neural and immune interactions

### 3.1 Dynamic regulation of the local immune microenvironment

Beyond functioning as a metabolic hub, BAT constitutes a highly dynamic niche for immunometabolic crosstalk. Crucially, the M1-to-M2 phenotypic transition of BAT-resident macrophages bidirectionally modulates local thermogenesis and microenvironmental immune tolerance. During healthy homeostasis and adaptive thermogenesis, the expansion of anti-inflammatory M2 macrophages is a core pathway that promotes the browning of WAT and maintaining BAT’s metabolic adaptability (24, 34). The specific chemokine CXCL13 can directionally recruit M2 macrophages, significantly suppressing local sterile inflammatory responses and thereby ensuring the smooth progression of adaptive thermogenesis (76). Furthermore, macrophages exhibit a high degree of tissue heterogeneity; for example, the Rarres2-positive macrophage subpopulation activates the AKT-mTOR signalling pathway within adipocytes via the Rarres2-Cmklr1 receptor axis, thereby facilitating the differentiation of BAT progenitor cells into mature adipocytes and upregulating the expression of key thermogenic proteins (67). The macrophage secreted phospholipase PLA2G2D similarly promotes browning of adipocytes and alleviates local inflammation (77). At the level of intercellular metabolic communication, active thermogenic adipocytes package damaged mitochondria into EVs for release (38), whilst resident macrophages actively participate in local microenvironmental mitochondrial quality control by phagocytosing these exosomes, a process that is crucial for maintaining the normal physiological function of BAT (78).

However, under metabolic stress conditions such as obesity, the pathological expansion of adipose tissue disrupts this immune balance, leading to massive infiltration of pro inflammatory M1 macrophages and other immune cells. The microenvironment of high-fat diet-induced obesity disrupts the heparan sulphate receptors on the surface of macrophages, impairing their ability to uptake extracellular mitochondria and thereby blocking the aforementioned protective intercellular mitochondrial transfer network (65, 79). Oxidative stress and hypoxia in the microenvironment induce macrophage polarisation towards the M1 phenotype; these M1 macrophages activate hypoxia-inducible factor-1α (HIF-1α) within brown adipocytes(BAs) via paracrine pathways, potentially reducing their sensitivity to β-adrenergic stimulation and downregulating UCP1 expression (80). Concurrently, metabolic stress drives the recruitment of circulating monocytes into tissues via the CCR2 pathway, forming an inflammatory infiltration network. Some macrophages polarise into lipid-associated macrophages following lipid phagocytosis; by secreting transforming growth factor-β1, they induce surrounding adipocytes to suppress thermogenic genes, thereby mediating the loss of local thermogenic characteristics (69, 81). When microenvironmental imbalance leads to excessive thermogenic stress, BAs actively release adenosine triphosphate (ATP) into the microenvironment as an alarm signal, activating the purinergic receptors P2X4 and P2X7 on the surface of macrophages. This further establishes a pro-inflammatory adipocyte macrophage communication axis and accelerates tissue functional decline (82). In addition to macrophages, ageing and obesity drive the abnormal accumulation of T-bet-expressing B cells in adipose tissue, which release the chemokine CXCL10, thereby exacerbating local sterile inflammation (83).

Beyond macrophages, the complement system, in conjunction with other resident lymphocytes, maintains BAT’s immune privilege and tissue remodelling. Adipsin, a complement protein specifically secreted by adipocytes, negatively regulates thermogenesis via an autonomous pathway; blocking its homologous receptor C3aR1 results in significantly enhanced maximal mitochondrial respiratory capacity and uncoupling metabolism in adipocytes (84). More crucially, a distinct population of regulatory T cells(Tregs) resides within BAT; cold stimulation selectively expands a subset of Tregs expressing IL-18R1. By suppressing the production of interferon-γ by local lymphocytes, these cells rigorously protect BAs from immune-mediated mitochondrial dysfunction, acting as an immune barrier that maintains thermogenic energy expenditure and cold tolerance (85). Concurrently, microvascular endothelial cells and their remodelling are deeply involved in the local immune microenvironment. Through mechanisms such as the release of extracellular adenosine (86) or the downregulation of SIRT3 (87), the vascular endothelium regulates lipid metabolism in peripheral adipocytes and influences the pro-inflammatory polarisation and infiltration of macrophages.

### 3.2 Sympathetic-endocrine-immune cross-talk

The homeostasis of the BAT microenvironment is highly dependent on the sympathetic-endocrine-immune reflex arc, centred on the brain-adipose axis (**Figure 1**). This central regulatory network originates in the hypothalamus; for example, neurons expressing pre enkephalin in the dorsal region of the ventromedial hypothalamic nucleus act as a cold sensitive centre. Upon sensing cold, these neurons are activated and rapidly increase BAT thermogenesis and core body temperature via the sympathetic nervous system (88). Concurrently, the activation of central leptin receptors (35) and oestrogen (89), via signals released by the hypothalamus, can enhance sympathetic innervation to BAT, thereby mediating cardioprotective effects and energy homeostasis; this central regulation is contingent upon an intact local sympathetic innervation network (90). Sympathetic nerve terminals act interact with on β3-adrenergic receptor (β3-AR) located on the surface of adipocytes and resident cells in the microenvironment by releasing noradrenaline. This not only drives intracellular triglyceride lipolysis and mitochondrial oxidative phosphorylation to generate heat (14), but also profoundly alters the local endocrine and immune communication landscape (91). At the cellular level, norepinephrine(NE) potently induces the expression of transcription factors such as Foxj3 (92) and GATA3 (93) to drive adaptive thermogenesis and activates the PRMT4-catalysed cascade (94), precisely translating systemic environmental stress into local signals for adipose remodelling. Furthermore, β3-AR signalling can inhibit the expression of pro-inflammatory mediator-induced nitric oxide synthase and its accumulation in exosomes via the extracellular signal-regulated kinase pathway, demonstrating the sympathetic nervous system’s ability to finely regulate endocrine cargo (95).

**Figure 1:**
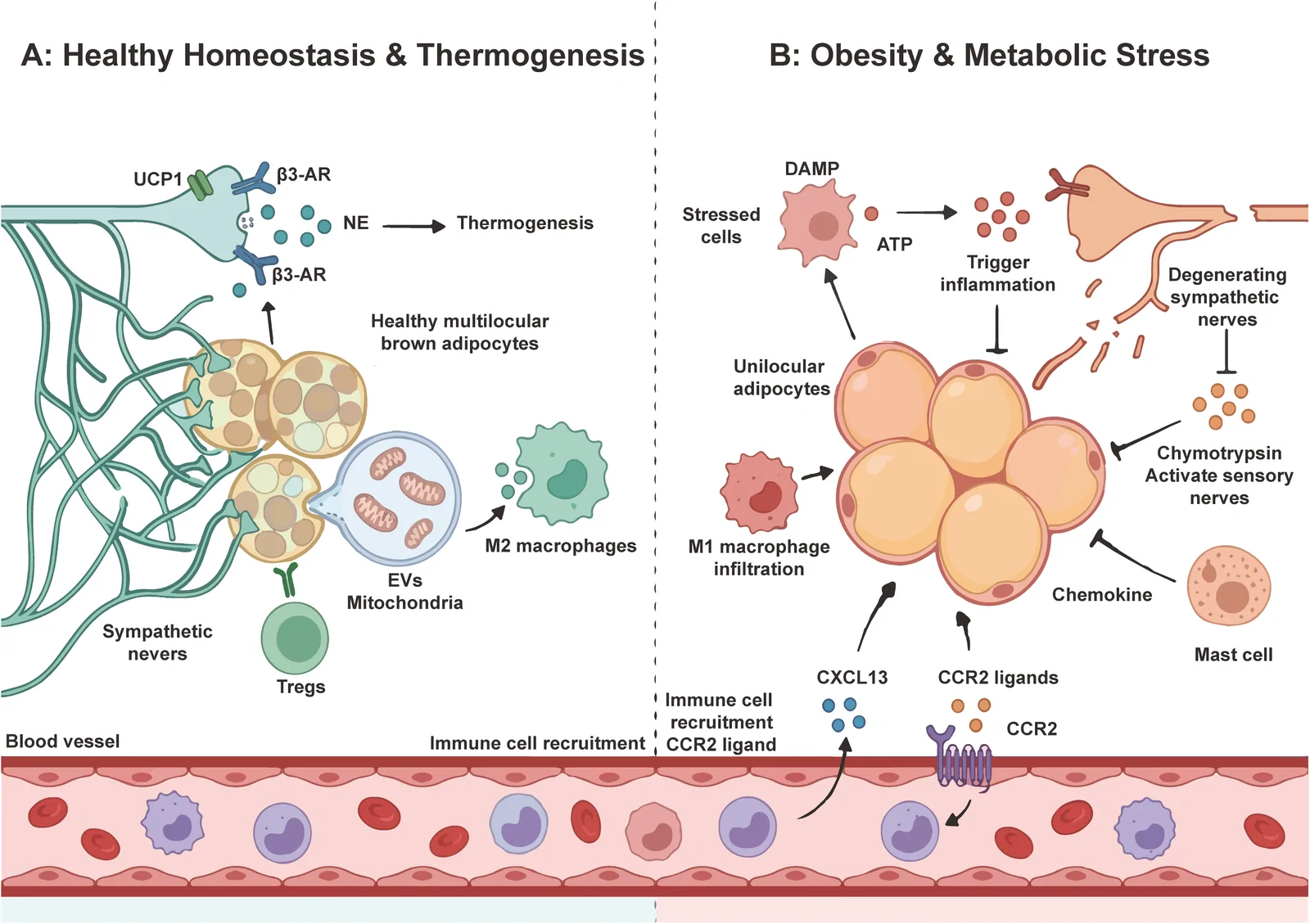
BAT immune microenvironmental remodeling in health and obesity. **(A)** Healthy Homeostasis and Thermogenesis. Sympathetic nerve terminals release NE to engage β3-AR on mitochondria-rich, multilocular BAs. Consequently, NE signaling activates inner mitochondrial membrane-localized UCP1, uncoupling oxidative phosphorylation to drive thermogenesis. Concurrently, resident anti-inflammatory M2 macrophages and Tregs maintain tissue homeostasis by suppressing pro-inflammatory cascades. Furthermore, a robust microvasculature supports tissue integrity. Crucially, BAs package mitochondria into EVs for intercellular transfer to M2 macrophages, fundamentally sustaining immunometabolic crosstalk. **(B)** Obesity and Metabolic Stress. Conversely, chronic metabolic stress and obesity precipitate detrimental BAT microenvironmental remodeling. The tissue exhibits marked infiltration by hypertrophic unilocular adipocytes alongside pro-inflammatory populations including mast cells and M1 macrophages. Stressed adipocytes subsequently release DAMPs including ATP to trigger localized inflammation. Moreover, these activated adipocytes secrete chemokines including CXCL13 and CCR2 ligands into the microcirculation. Concomitantly, sympathetic nerve terminals undergo profound degeneration and fragmentation. Furthermore, pro-inflammatory mast cells secrete proteases including chymotrypsin to abrogate thermogenesis via the cleavage of neural or thermogenic signaling components. Simultaneously, CCR2 signaling recruits circulating monocytes from the microvasculature, driving their differentiation into pathogenic M1 macrophages. Ultimately, global suppression of thermogenic capacity and exacerbated microenvironmental inflammation occur relative to physiological homeostasis. ATP, Adenosine triphosphate; BAs, Brown adipocytes; BAT, Brown adipose tissue; β3-AR, β3-adrenergic receptor; CCR2, C-C chemokine receptor type 2; CXCL13, C-X-C motif chemokine ligand 13; DAMP, Damage-associated molecular pattern; EVs, Extracellular vesicles; NE, Norepinephrine; Treg, Regulatory T cell; UCP1, Uncoupling protein 1.

Even more ingeniously, the highly dense network of sympathetic synapses within BAT is precisely sculpted and maintained by local target cells and immune cell populations. Adipocytes actively secrete neurotrophic factors such as neurotrophin-4 (41), neurotrophin 3 (96) and S100B (97), which potently promote the axonal outgrowth of sympathetic nerve fibres, thereby constructing and maintaining a dense network of sympathetic innervation. Paracrine signals from specific immune cells are also involved in guiding the ingrowth of sympathetic terminals; for example, γδ T cells within adipose tissue secrete interleukin-17 (98), which stimulates BAs to produce transforming growth factor-β. This immune-metabolic cascade ultimately sustains the local projection of sympathetic nerve fibres (99). Vascular smooth muscle cells(VSMCs) act as neuroimmune relay stations in this process; their specific secretion of the chemokine Cxcl12 not only actively recruits macrophages but also simultaneously maintains the microenvironmental architecture of sympathetic ganglia and the normal release of noradrenaline (100). Sympathetic ganglion cells, acting as key physical hubs for maintaining the neuro-tissue barrier, secrete interleukin-33 during cold exposure. Through matrix-immune crosstalk, they sustain the survival of Tregs and eosinophils in the microenvironment, which is crucial for the compensatory upregulation of downstream thermogenic genes (101). At the local negative feedback level, thermogenic stimulation induces BAT to upregulate the expression of the kininogen and bradykinin systems, interfering with the kinase signalling pathways that control core thermogenesis and providing highly active BAT with endocrine braking signals to prevent excessive energy expenditure (102). Furthermore, mast cells in the microenvironment activate protease-activated receptors on adjacent sensory nerve fibres by releasing granules containing chymotrypsin, forming a tissue-spanning retrograde immuno-neural direct communication pathway that rapidly transmits signals to the central nervous system during stress states such as shock, thereby strongly inhibiting BAT thermogenesis (103).

In conditions of obesity, ageing or chronic sterile inflammation, this neuro-adipose-immune reflex arc is severely disrupted. A persistent inflammatory microenvironment not only correlates with the downregulation of the abundance of β-adrenergic receptors on the surface of adipocytes, impeding the trans-membrane conduction of catecholamine signalling, but also leads to a decline in the adipose tissue’s ability to secrete nerve growth factor 1, triggering degenerative changes in sympathetic nerve projections (57, 104). During the ageing process, S100A8-positive senescent immune cells originating from the bone marrow extensively infiltrate BAT. By inhibiting specific RNA-binding motif proteins, they contribute to the dysregulation of axon-guidance gene expression, thereby disrupting local sympathetic innervation (105). When the original neurally mediated thermogenic pathways are compromised, immune cells within the microenvironment exhibit a robust compensatory mechanism. Interleukin-6(IL-6), induced by exercise or acute stress (106), can activate eosinophils and induce macrophages to undergo M2 alternative polarisation. These polarised macrophages acquire the ability to synthesise tyrosine hydroxylase, enabling them to release NE locally, thereby synergistically amplifying sympathetic signals and even replacing damaged neurons to drive adipose remodelling (24, 107, 108). However, if peripheral neural signalling via 5-hydroxytryptamine is abnormally elevated or the endoplasmic reticulum regulatory protein REEP6 is severely deficient (109), the metabolic-neural crosstalk network within the microenvironment will undergo complete decompensation, ultimately accelerating the progression of fat depigmentation and cardiovascular metabolic diseases (110).

## 4 Mediators and pathways of BAT-mediated cross-organ regulation of cardiovascular immunity

### 4.1 Classical endocrine protein/peptide pathways

BAT is not only a high-energy thermogenic reservoir but also a highly active endocrine organ. Beyond its traditional adaptive thermogenic function, BAT is capable of specifically secreting and releasing large quantities of bioactive macromolecular peptides and proteins, known as Batokines (20), into the systemic circulation (111). Through cross-organ communication networks, these endocrine mediators extensively target the liver, skeletal muscle, the bone marrow immune microenvironment and the cardiovascular system, playing an irreplaceable systemic protective role in regulating whole-body energy homeostasis and cardiovascular immunity (19, 112, 113) (**Figure 2**). In-depth analysis of the extensive secretome profile of BAT has revealed its translational value as a novel molecular communication hub for the intervention of cardiovascular and metabolic diseases (23, 32, 114).

**Figure 2:**
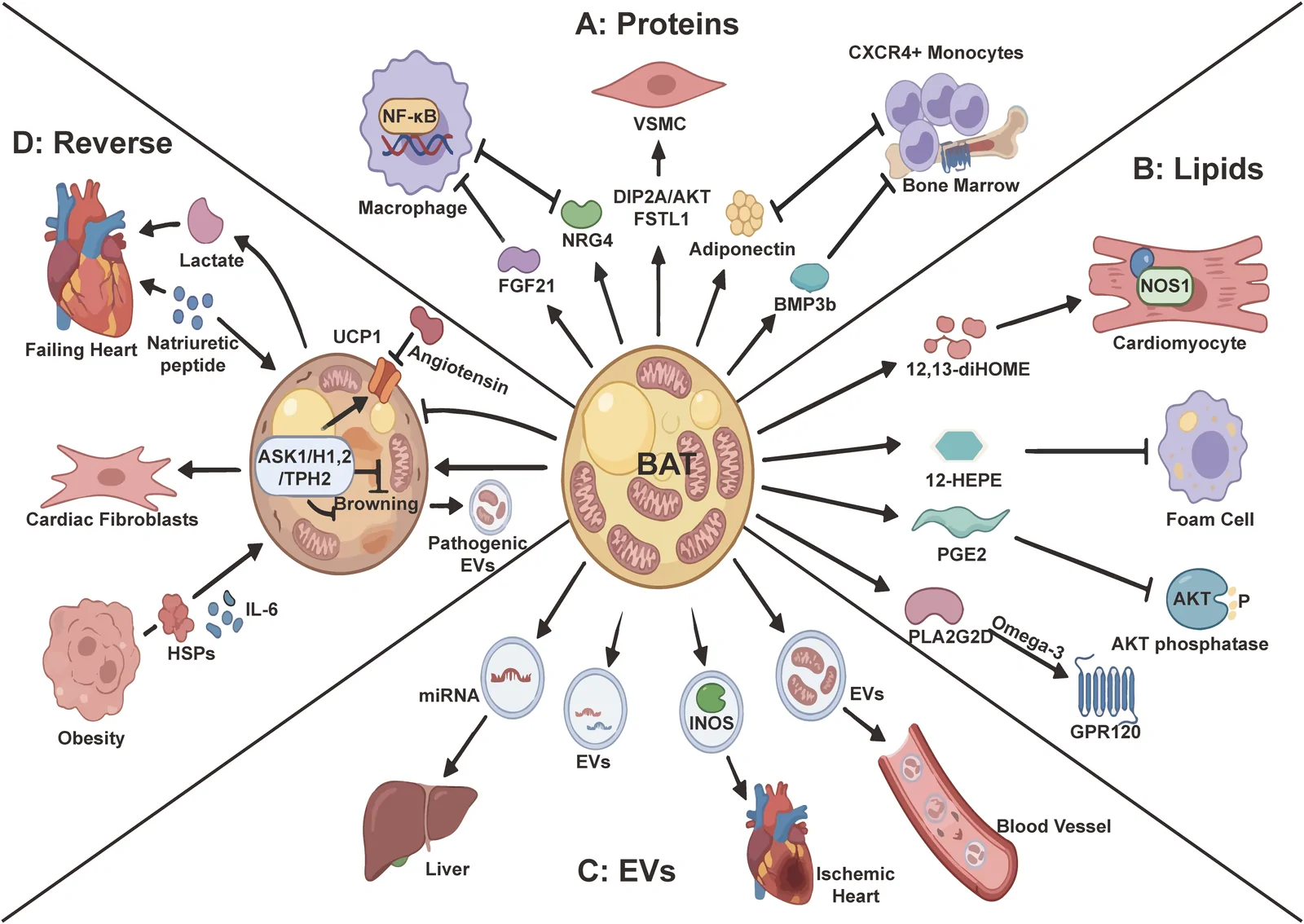
Mechanisms of BAT-mediated cross-organ immune regulation and reverse remodeling. BAT orchestrates systemic homeostasis and adapts to pathological stress via four primary mechanisms. **(A)** Endocrine Protein Pathways: BAT secretes batokines including FGF21, NRG4, FSTL1, adiponectin and BMP3b. Specifically, FSTL1 activates the VSMCs DIP2A/AKT pathway. Concurrently, FGF21 and adiponectin suppress macrophage NF-κB signaling, while BAT directly attenuates bone marrow CXCR4+ monocyte release. **(B)** Lipid-Mediated Pathways: BAT releases lipokines including 12,13-diHOME, 12-HEPE and PGE2. Mechanistically, 12,13-diHOME targets cardiomyocyte NOS1, 12-HEPE inhibits foam cell formation and PGE2 antagonizes AKT phosphatase. Furthermore, PLA2G2D mobilizes omega-3 to activate GPR120. **(C)** EV Communication: BAT-derived EVs deliver miRNAs to the liver and iNOS to ischemic myocardium. Pathologically, EVs encapsulating damaged mitochondria breach tissue barriers and enter the systemic circulation. **(D)** Reverse Stress Remodeling: Failing myocardium releases lactate and natriuretic peptides to stimulate BAT. Conversely, angiotensin abrogates BAT UCP1 function. Concomitantly, systemic stressors obesity-derived HSPs and IL-6 upregulate ASK1, H1. 2 and TPH2, intrinsically suppressing adipose browning. Consequently, stressed BAT secretes pathogenic EVs that directly accelerate cardiac fibroblast remodeling. 12,13-diHOME, 12,13-dihydroxy-9Z-octadecenoic acid; 12-HEPE, 12-Hydroxyeicosapentaenoic acid; AKT, Protein Kinase B; ASK1, Apoptosis Signal-regulating Kinase 1; BAT, Brown Adipose Tissue; BMP3b, Bone Morphogenetic Protein 3b; CXCR4, C-X-C Motif Chemokine Receptor 4; DIP2A, Disco-Interacting Protein 2 Homolog A; EVs, Extracellular Vesicles; FGF21, Fibroblast Growth Factor 21; FSTL1, Follistatin-like 1; GPR120, G-Protein Coupled Receptor 120; H1.2, Histone variant H1.2; HSPs, Heat Shock Proteins; IL-6, Interleukin-6; iNOS, Inducible Nitric Oxide Synthase; miRNAs, microRNAs; NF-κB, Nuclear Factor Kappa B; NOS1, Nitric Oxide Synthase 1/Neuronal NOS; NRG4, Neuregulin 4; PGE2, Prostaglandin E2; PLA2G2D, Phospholipase A2 Group IID; TPH2, Tryptophan Hydroxylase 2; UCP1, Uncoupling Protein 1; VSMC, Vascular Smooth Muscle Cell.

Among numerous endocrine peptides, fibroblast growth factor 21 (FGF21) and neuroregulatory protein 4 (NRG4) are the core, classic batokines mediating cardiovascular immune regulation. In response to cold stress or adrenergic stimulation, BAs extensively transcribe and secrete FGF21 into the bloodstream (115). Circulating FGF21 can target cardiac and skeletal muscle, enhancing energy metabolism by promoting the formation of mitochondrial cristae within target cells and demonstrating potent anti-fibrotic and anti-hypertrophic effects (2, 116, 117). At the level of immune regulation, FGF21 can specifically activate the NF-κB pathway in peripheral macrophages and potently inhibit the NF-κB inflammatory signalling cascade, thereby attenuating macrophage-mediated sterile inflammation of the vascular wall (118). Furthermore, FGF21 acts as a key cross-organ messenger that mediates adenosine receptor activation to antagonise hypertensive ventricular remodelling (1). Meanwhile, NRG4, which is highly enriched in BAT, acts as another key endocrine factor and is significantly downregulated in the circulation of obese individuals (41). Actively secreted NRG4 not only drives the systemic production of a healthy spectrum of adipokines but also enters the circulation to modulate regulate the macrophage polarisation network in distally damaged tissues (26) and inhibits the pathogenic functions of myofibroblasts, thereby exerting immunometabolic protective effects against AS and promoting tissue repair (119).

In addition to FGF21 and NRG4, other bioactive proteins secreted by BAT also exert synergistic effects on the immunostatic balance of the cardiovascular microenvironment. For example, follicle-stimulating hormone-like protein 1 (FSTL1) has been identified as a novel vasoprotective endocrine factor; upon release into the bloodstream, it targets the vascular wall and, by specifically activating the DIP2A/AKT signalling pathway, effectively inhibits apoptosis in VSMCs, thereby preventing the development of abdominal aortic aneurysms (120). Bone morphogenetic protein 3b (BMP3b) (121) and nerve growth factor (117) act as potential endocrine protective factors in myocardial ischaemia reperfusion injury, promoting cardiomyocyte survival. As a classic adipokine, adiponectin not only promotes the proliferation of M2 anti-inflammatory macrophages and AKT activation in target tissues following cold exposure, but the restoration of its systemic circulating levels can also reverse cardiovascular inflammatory damage, protecting blood vessels from the progression of atherosclerotic plaques (122) and aneurysms (111, 123).

BAT-mediated endocrine regulation can also transcend spatial barriers, engaging in profound cross-organ dialogue with the bone marrow haematopoietic and immune systems. Preclinical studies indicate that ambient temperature and BAT activity profoundly influence the kinetics of extramedullary monocyte recruitment. In a thermoneutral environment lacking BAT thermogenic activation, CXCR4 positive monocytes accumulate extensively within the bone marrow; their bone marrow extravasation, which depends on the CCR2 signalling pathway, is significantly inhibited, thereby reducing at the source the number of pathogenic monocytes in the circulatory system and their inflammatory recruitment into atherosclerotic plaques (124). Furthermore, the circulating factor CXCL14, which possesses immunoregulatory properties, also plays a key endocrine signalling role in metabolic networks and fat distribution during early life (125). This suggests that BAT and its endocrine network can exert an upstream regulatory role in systemic cardiovascular immune homeostasis by modulating the release thresholds and peripheral polarisation fate of bone marrow immune cells.

### 4.2 Lipid-mediated signalling pathways

BAT not only exerts endocrine functions through the secretion of polypeptides but also plays a central role in cross-organ communication by releasing specific lipid mediator molecules (lipokines) (**Figure 2B**). These lipid mediators demonstrate potent targeted effects in attenuating vascular inflammation, improving cardiovascular haemodynamics and regulating systemic immune-metabolic networks (113). When stimulated by cold or exercise, BAT specifically releases the lipid molecule 12,13-diHOME into the systemic circulation (116). Circulating 12,13-diHOME directly targets cardiomyocytes and, via a nitric oxide synthase 1 mediated mechanism, significantly increases cardiac mitochondrial respiration, thereby enhancing cardiac pumping efficiency and improving haemodynamic functions such as systole and diastole (126). BAT transplantation experiments have also confirmed that the release of 12,13-diHOME can directly improve the physiological function of the targeted damaged heart (2). Furthermore, vascular endothelial nitric oxide signalling can induce an increase in BAT mass, accompanied by a significant rise in plasma 12,13-diHOME synthesis, indicating the existence of a highly integrated lipid metabolic cascade between the adipose and cardiovascular systems (9).

In terms of immunoregulation and anti-AS, specific hydroxyeicosatetraenoic acid derivatives released by BAT play a key role. BAT-specific 12-lipoxygenase catalyse the production of 12-HEPE; this lipid molecule not only promotes glucose uptake in target organs by activating the PI3K-mTOR-Akt-GLUT pathway (116), but also enters the circulation to target macrophages in the vascular wall, effectively inhibiting excessive cholesterol accumulation and the formation of foam cells, thereby improving the cardiovascular pathological state induced by a high-fat diet (20). Conversely, obesity is often accompanied by pathologically elevated levels in the circulation of the pro-inflammatory lipid mediators 5-hydroxy-eicosatetraenoic acid and 11-hydroxy-eicosatetraenoic acid, which are derived from arachidonic acid (127). Targeted knockout of 12-lipoxygenase and its downstream lipid mediators confers significant resistance to Western diet-induced high-fat obesity and systemic immune inflammation, highlighting the central role of this lipid pathway in regulating the pathological polarisation of macrophages (127).

In mediating the resolution of inflammation, BAT also relies on specific pro-anti-inflammatory mediators and the metabolic network of omega-3 polyunsaturated fatty acids (127). Cold exposure and sympathetic stimulation promote the production of Maresin 2 in BAT, a highly specific pro-anti-inflammatory mediator that primarily targets macrophage populations in microenvironments such as the liver via cross-organ mechanisms, thereby significantly alleviating obesity-induced systemic chronic inflammation (128). Furthermore, the secretory phospholipase PLA2G2D plays a metabolically protective role in mobilising tissue-bound omega-3 polyunsaturated fatty acids; as a key enzyme upstream of the free fatty acid receptor GPR120, it promotes targeted anti-inflammation and lipobrowning by providing endogenous omega-3 precursors (77). Targeted supplementation with these marine-derived precursors increases plasma levels of specific pro-anti-inflammatory mediators, thereby improving inflammation and insulin resistance in the cardiovascular system and liver (129, 130). Palmitoleic acid secreted by WAT also effectively reduces inflammation in target organs and decreases ectopic lipid accumulation in the local microenvironment by increasing insulin receptor expression in target cells (116).

Prostaglandins and their associated metabolic pathways constitute another important pillar of BAT lipid signalling (131). BAT is capable of specifically secreting prostaglandin E2 and prostaglandin F2α; these lipid factors act as potent endocrine insulin sensitizers by inhibiting specific AKT phosphatases, thereby protecting the body from metabolic damage (132). This process is strictly epigenetically regulated by the m6A methyltransferase METTL14, which limits the release of these beneficial lipid factors by accelerating the degradation of prostaglandin core enzyme transcripts (132). There is also profound functional synergy between microsomal prostaglandin E synthase-1 and the nuclear receptor PPARγ, which critically regulates the homeostasis of prostaglandin conversion and activity (129). Furthermore, endocrine factors such as FGF6 and FGF9 induce the expression of thermogenic genes by upregulating the biosynthesis and release of prostaglandin E2, utilising it as a key lipid intermediate (133). Furthermore, endocrine factors such as FGF6 and FGF9 induce the expression of thermogenic genes by upregulating the biosynthesis and release of prostaglandin E2, utilising it as a key lipid intermediate (55).

### 4.3 Exosome and miRNA-mediated epigenetic communication

The cross-organ communication network of BAT relies not only on soluble peptides and free lipids; epigenetic information transfer, dominated by EVs and particularly exosomes, is emerging as a core mechanism regulating cardiovascular immunity and systemic metabolism (134) (**Figure 2C**). BAT is capable of secreting large quantities of small EVs into the circulatory system; these vesicles are rich in mitochondrial functional components, specific proteins and miRNAs, acting as long-distance communication carriers that directly target the distal cardiovascular system and other metabolic target organs (135, 136). Upon activation by thermogenic stimuli such as cold or exercise, BAT promotes the massive release of these vesicles, precisely packaging specific epigenetic regulatory factors within them (137, 138). For example, BAT exosomes are rich in specific miRNA molecules, namely miR-99b and miR-378a-3p. These vesicles, carrying regulatory instructions, not only cross organ barriers to enhance hepatic gluconeogenesis to meet energy demands, but also, upon uptake by target organs, directly reshape the metabolic and inflammatory gene expression networks within recipient cells at the post-transcriptional level (113).

In terms of cardiovascular protection and immunomodulation, BAT-derived exosomes have demonstrated significant potential for targeted therapy. Exercise-induced BAT exosomes are capable of transporting specific miRNAs via the bloodstream to directly regulate the survival of distally ischaemic or damaged cardiomyocytes, thereby exerting a crucial cross-organ cardioprotective effect (138). More importantly, BAT exosomes can also serve as carriers for specific enzymes to directly intervene in the cardiac microenvironment. Encapsulating iNOS within BAT-derived exosomes and delivering it specifically to the heart can significantly reverse exercise-induced pathological cardiac remodelling (95). In models of obesity or metabolic syndrome, systemic infusion of healthy BAT exosomes not only improves overall insulin sensitivity but also effectively reduces localised cardiac lipid deposition, partially restoring ventricular function impaired by a high-fat diet (2, 135). Furthermore, BAT-derived small EVs can specifically load and deliver the adipokine phospho-nuclear protein 3, which targets and regulates the mRNA stability of key thermogenic transcription factors in peripheral tissues, inducing anti-inflammatory browning reprogramming of the adipose immune microenvironment (136).

Beyond nucleic acids and proteins, adipocyte-derived EVs are also deeply involved in the cross-organ transport of pathological mitochondria. When excessive thermogenic stress causes BAs to release large quantities of vesicles containing damaged mitochondrial fragments to maintain their own homeostasis (38, 78), if the body is in a metabolic microenvironment such as obesity, the phagocytic clearance function of local macrophages will undergo the dysregulation and collapse described earlier. The failure of this local defence mechanism directly results in a large number of fat-derived mitochondrial vesicles crossing tissue boundaries and entering the systemic circulation, thereby transforming into cross-organ pathogenic carriers that trigger systemic metabolic disorders and immune disturbances (65).

### 4.4 Cardiovascular stress-induced reverse immunometabolic remodelling of BAT

Cross-organ communication between BAT and the cardiovascular system is not a unidirectional process, but rather a complex bidirectional interactive network (**Figure 2D**). When the cardiovascular system encounters severe pathogenic stress, systemic signals generated by the affected organs trigger a reciprocal remodelling of the BAT microenvironment. In non reperfusion myocardial infarction (121) and the early stages of myocardial hypertrophy induced by transverse aortic constriction (139), severe ischaemic injury and pathological stress in the heart trigger cross-organ, intense compensatory activation of peripheral BAT and browning of subcutaneous fat; this process is accompanied by cardiac secretion of natriuretic peptides promoting energy dissipation in BAT (108). Furthermore, the large amounts of lactate released due to cardiac dysfunction-induced inactivation of cardiac pyruvate dehydrogenase can also promote cross-organ browning and remodelling of peripheral adipose tissue (140). However, this early cardiac stress-driven compensatory adaptation of fat metabolism is not sustainable. As chronic heart failure progresses, this compensatory mechanism undergoes a complete reversal, manifested by a dramatic downregulation of oxidase enzymes within BAT and severe systemic insulin resistance (139). In the late stages of severe heart failure, accompanied by extreme impairment of left ventricular systolic function, markedly elevated sympathetic tone leads to pathological overactivation of BAT,The failing heart tends to rely on glucose for energy substrate utilisation, thereby competing with highly active BAT for circulating glucose substrates, further exacerbating cardiac dysfunction until the BAT-induced thermogenic compensatory mechanism fails completely, resulting in fatal hypothermia (141).

In addition to the metabolic shift triggered by heart failure, the angiotensin-mediated stress network associated with CVD can also disrupt the immunometabolic homeostasis of BAT. Angiotensin-mediated endothelial dysfunction blocks the compensatory upregulation of cold-induced UCP1 and mitochondrial respiratory chain complexes at the transcriptional level, completely negating the body’s thermogenic protective mechanisms (9). More seriously, when endogenous protective receptors in the cardiovascular system are compromised, pathological angiotensin stress induces dysfunctional BAT to release pathogenic exosomes. Once these abnormal vesicles are taken up by cardiac fibroblasts, they conversely accelerate their pathological transformation into myofibroblasts, significantly exacerbating local cardiac fibrosis and the remodelling of inflammatory gene expression (142). Furthermore, the GRK2 N-terminal peptide, which is specifically expressed by the heart under stress, can induce systemic degenerative changes in BAT metabolic signalling and thermogenic capacity via cross-organ communication (143).

Systemic metabolic dysregulation and severe systemic stress are equally central factors driving the pathological remodelling of BAT. Under conditions of severe metabolic stress, such as tumour cachexia, heat shock proteins, parathyroid hormone-related proteins and IL-6 secreted by malignant tissues hijack the body’s thermogenic network, forcing the mitochondria of fat cells to undergo abnormal oxygen consumption polarisation and an increase in basal metabolic rate (144, 145). This forced pathological browning and extreme energy-consuming state accelerate systemic lipid mobilisation and immune-metabolic failure, creating a vicious cycle that exacerbates disease progression (107, 146). Concurrently, chronic stressors such as long-term obesity and high-fat diets induce a highly conserved reverse-whitening remodelling of classical BAT and perivascular fat (147), manifested as a reduction in interstitial microcirculation, a precipitous decline in the maximum diet-induced thermogenic threshold and abnormal local differentiation towards a fibrotic phenotype (6, 46). In the obese microenvironment, excessive accumulation of visceral fat acts as a toxic reservoir that depletes circulating natriuretic peptide levels (148), whilst adipocytes upregulate the expression of inhibitory factors such as the stress kinase ASK1 (149), the histone variant H1. 2 (150) and TPH2 (110), thereby actively blocking the compensatory browning thermogenic network. This collapse of the local BAT immune-inflammatory network, triggered by external cardiovascular or systemic metabolic stress, not only terminates BAT’s original endocrine protective function but also transforms it into an amplifier of systemic chronic inflammation, thereby driving a vicious cycle of cardiovascular complications.

### 4.5 Targeted regulation of cardiac immune cell populations by BAT-derived signals under pathological stress

BAT profoundly orchestrates the cardiac immune microenvironment through the targeted delivery of specific endocrine, lipid, and epigenetic signals during cardiovascular pathogenesis (**Table 2**). Specifically, under conditions of hypertensive cardiac remodeling and ischemic injury, BAT-derived FGF21 acts as a critical immunomodulator. Circulating FGF21 directly targets peripheral macrophages to potently suppress the NF-κB inflammatory signaling cascade, thereby attenuating macrophage-mediated sterile inflammation within the myocardium and vascular wall (1, 118). Furthermore, restoring adipocyte leptin signaling enhances BAT thermogenesis and subsequently reduces localized tissue inflammation and macrophage infiltration in the heart (151).

**Table 2:** Overview of BAT-derived signals targeting specific immune cell populations in cardiovascular pathologies.

BAT-derived signal	Target immune cell population	Associated cardiovascular pathology	Immunological mechanism	References
Thermogenic Status	CXCR4+/CCR2+ Monocytes	AS	Modulates bone marrow extravasation; BAT inactivation restricts pathogenic monocyte circulation and subsequent recruitment into atherosclerotic lesions	(124)
FGF21	Peripheral Macrophages	Hypertensive Cardiac Remodeling	Suppresses the NF-κB inflammatory cascade to attenuate vascular sterile inflammation and reverse cardiac fibrosis	(1)
NRG4	Macrophages & Myofibroblasts	AS & Tissue Injury	Orchestrates macrophage polarization and mitigates pathogenic myofibroblast activity to facilitate tissue repair	(119)
Adiponectin	M2 Macrophages	AS & Vascular Inflammation	Stimulates anti-inflammatory M2 macrophage proliferation to resolve cardiovascular inflammatory damage	(111)
12-HEPE	Vascular Macrophages	High-Fat Diet-Induced AS	Impedes aberrant cholesterol accumulation and precludes foam cell formation within the vascular wall	(116)
iNOS	Cardiac Microenvironment	Sympathetic Stress-Induced Cardiac Remodeling	Mediates targeted iNOS delivery to the myocardium, thereby rescuing exacerbated cardiac remodeling phenotypes	(95)

12-HEPE, 12-Hydroxyeicosapentaenoic acid; AS, Atherosclerosis; BAT, Brown Adipose Tissue; CCR2, C-C Motif Chemokine Receptor 2; CXCR4, C-X-C Motif Chemokine Receptor 4; FGF21, Fibroblast Growth Factor 21; iNOS, Inducible Nitric Oxide Synthase; NF-κB, Nuclear Factor Kappa B; NRG4, Neuregulin 4.

In addition to peptide hormones, BAT releases specific lipid mediators to dictate macrophage polarization. Cold-induced secretion of Maresin 2 serves as a specialized pro-resolving mediator that selectively targets macrophage populations to mitigate systemic chronic inflammation (128). Similarly, the BAT-specific lipokine 12-HEPE directly targets vascular macrophages, effectively impeding aberrant cholesterol accumulation and precluding foam cell formation within the vascular wall (116). Concurrently, adiponectin secreted by BAT stimulates the proliferation of anti-inflammatory M2 macrophages via AKT pathway activation, actively resolving local cardiovascular inflammation (111). Moreover, the brown fat-enriched hormone NRG4 enters the systemic circulation to meticulously regulate the macrophage polarization network and myofibroblast function in distally damaged tissues, facilitating cardiac repair (119).

Beyond local macrophage modulation, BAT activity systemically governs immune cell mobilization from the bone marrow to the cardiovascular system. Environmental thermoneutrality, which inactivates BAT, paradoxically causes the accumulation of CXCR4-positive monocytes within the bone marrow. This retention directly reduces the recruitment of pathogenic monocytes into atherosclerotic lesions and ischemic myocardium (124). At the epigenetic level, BAT-derived exosomes function as precise intercellular delivery vehicles. During pathological cardiac remodeling, BAT actively packages and transfers iNOS via exosomes directly to the myocardium, thereby rescuing the heart from sympathetic stress-induced hypertrophic deterioration (95). Additionally, localized crosstalk between adipose tissue and adaptive immune cells is evident, as γδ T cells drive the expression of transforming growth factor-β1 through IL-17RC signaling to regulate regional innervation and metabolic homeostasis (98). Consequently, harnessing this precise BAT-immune crosstalk provides a transformative therapeutic strategy to attenuate cardiac inflammation in advanced cardiometabolic diseases.

## 5 Mechanisms of BAT-targeted immunological interventions for specific CVD

Prior to delineating BAT immunomodulation in CVDs, outlining the evolving paradigm of BAT-targeted therapies across three specificity levels remains imperative. First, systemic agents including extracellular inosine (152) and CRISPR-activated FGF21/FNDC5 (153) broadly stimulate BAT thermogenesis and induce compensatory WAT browning. To circumvent subsequent systemic toxicity, precision delivery platforms utilizing adipose-targeted triiodothyronine selectively localize to brown and beige adipocytes, halting atherosclerosis without off-target effects (154). Furthermore, anatomical depot-specific interventions harness unique paracrine microenvironments. Procedures including thoracic perivascular adipose tissue transplantation for aortic aneurysms (123) and epicardial application of BAT-derived stem cell patches for myocardial infarction (155) precisely modulate localized immunological niches. Consequently, the mechanisms delineated hereafter encompass this comprehensive therapeutic spectrum, transitioning seamlessly from systemic metabolic modulation to depot-specific immune microenvironment resolution.

### 5.1 Immune responses of BAT in AS

The pathological core of AS lies in abnormal lipid deposition and chronic aseptic vascular inflammation, whilst the activation of BAT establishes a key defence against this pathological process by reshaping lipid metabolism and cross-organ immune networks (156) (**Figure 3A**). Studies have shown that the presence of active BAT in the human body not only independently and significantly reduces the risk of coronary heart disease and cerebrovascular disease, but its high glucose metabolic activity is also directly associated with significant improvements in subclinical AS indicators, such as reduced carotid intima-media thickness and decreased uptake of arterial wall inflammatory markers (79, 157). This vasoprotective effect of BAT is largely attributed to its substantial non-shivering thermogenic energy expenditure. Activated brown and beige adipocytes consume large amounts of circulating substrates, promoting the lipolysis of triglyceride-rich lipoproteins in plasma, thereby accelerating the clearance of very low-density lipoprotein (VLDL) remnants and depriving plaque formation of its lipid substrate at the source (18, 158). Concurrently, the lipoprotein remnants generated by BAT lipolysis can transfer excess surface phospholipids to newly formed high-density lipoproteins, thereby significantly enhancing the efficiency of cholesterol reverse transport within macrophages. This further inhibits the formation of foam cells within the vascular wall and limits the expansion of the necrotic core of the plaque (18).

**Figure 3:**
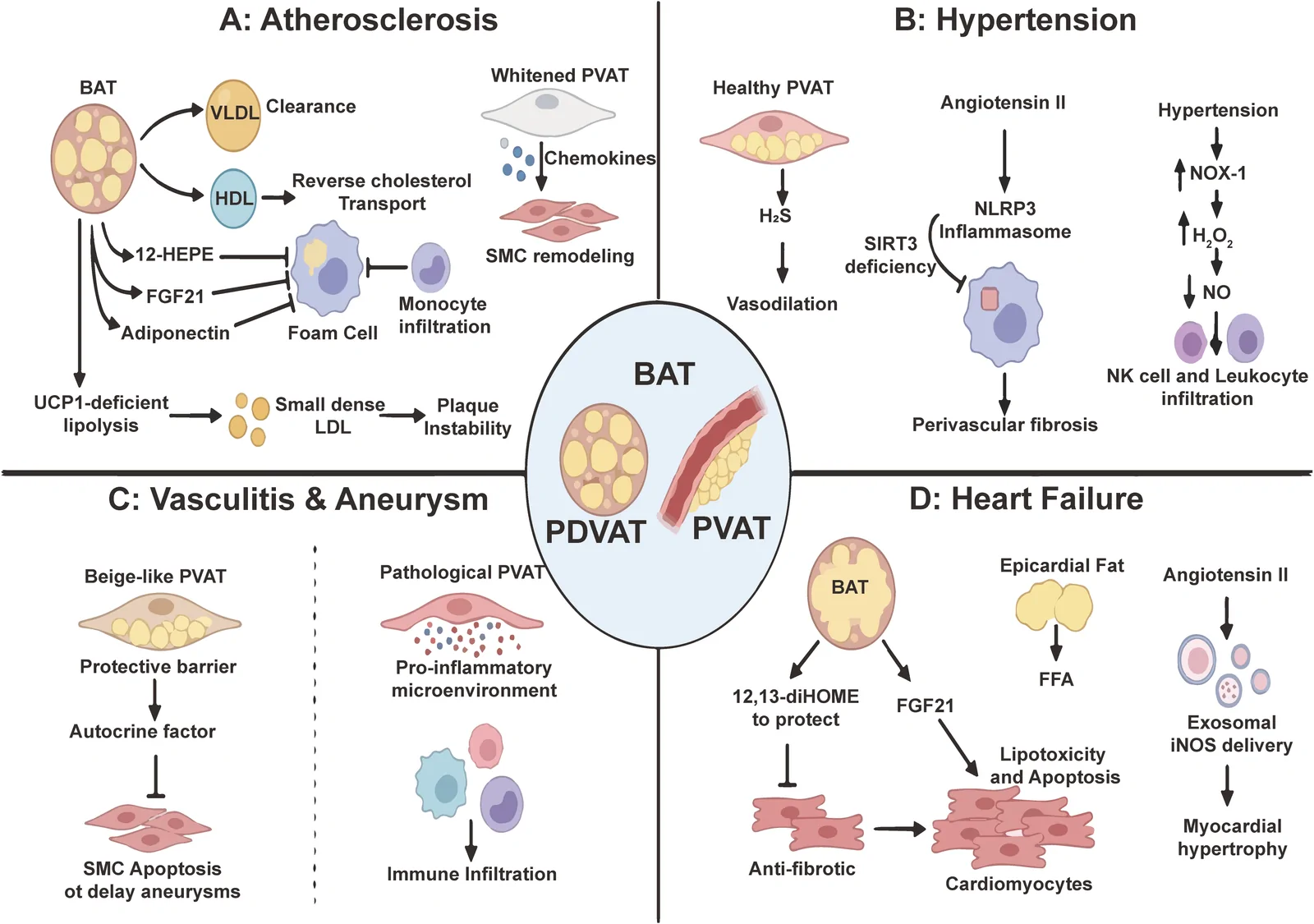
Mechanisms of BAT and PVAT-targeted immunological interventions in specific CVD. BAT and PVAT orchestrate dual immunometabolic regulation across four primary cardiovascular pathologies. **(A)** Atherosclerosis: BAT accelerates VLDL clearance and augments macrophage reverse cholesterol transport. Concurrently, endocrine factors including 12-HEPE, FGF21 and adiponectin suppress foam cell formation and monocyte infiltration. Conversely, UCP1-deficient lipolysis generates small dense low-density lipoproteins. This mechanism, alongside PVAT whitening, profoundly exacerbates plaque instability. **(B)** Hypertension: Physiological PVAT secretes hydrogen sulfide to maintain vasodilation. Pathologically, angiotensin II triggers macrophage SIRT3 deficiency, activating the NLRP3 inflammasome to precipitate perivascular fibrosis. Furthermore, hypertensive stress upregulates NOX-1 and hydrogen peroxide while diminishing nitric oxide bioavailability, orchestrating leukocyte and natural killer cell recruitment. **(C)** Vasculitis and Aneurysms: Beige-like PVAT functions as an immunological barrier, secreting autocrine factors that inhibit VSMCs apoptosis. Conversely, pathological PVAT establishes a pro-inflammatory microenvironment driving localized immune infiltration. **(D)** Heart Failure: BAT-secreted 12,13-diHOME and FGF21 confer potent cardioprotection. Conversely, EAT releases free fatty acids (FFA) to induce myocardial lipotoxicity. Concomitantly, angiotensin II-mediated stress provokes aberrant exosomal iNOS delivery, directly exacerbating myocardial hypertrophy. 12,13-diHOME, 12,13-dihydroxy-9Z-octadecenoic acid; 12-HEPE, 12-Hydroxyeicosapentaenoic acid; BAT, Brown Adipose Tissue; EAT, epicardial adipose tissue; FFA, Free Fatty Acids; FGF21, Fibroblast Growth Factor 21; H2O2, Hydrogen Peroxide; H2S, Hydrogen Sulphide; HDL, High-Density Lipoprotein; iNOS, Inducible Nitric Oxide Synthase; NK cell, Natural Killer cell; NLRP3, NLR Family Pyrin Domain Containing 3; NO, Nitric Oxide; NOX-1, NADPH Oxidase 1; PVAT, Perivascular Adipose Tissue; SIRT3, Sirtuin 3; SMC, Smooth Muscle Cell; UCP1, Uncoupling Protein 1; VLDL, Very Low-Density Lipoprotein.

At the level of endocrine and immune intervention, factors secreted by BAT directly contribute to the resolution of the inflammatory microenvironment within the vascular wall. The lipid metabolite 12-hydroxy-eicosapentaenoic acid released by BAT targets macrophages, directly inhibiting their foaming and slowing the progression of plaques induced by a high-fat diet (20). The endocrine peptide FGF21 not only accelerates lipid turnover by enhancing adipose tissue thermogenesis (159), but also, in conjunction with factors such as adiponectin, blocks the vascular wall infiltration of monocytes by suppressing the systemic inflammatory network (2). In contrast, abnormally elevated circulating levels of B-cell activating factor in states of metabolic dysregulation correlate positively with markers of early vascular endothelial damage, such as von Willebrand factor, suggesting that an imbalance in immune mediators accelerates endothelial cell adhesion and pathological monocyte chemotaxis (160).

Within the local vascular microenvironment, the phenotypic transformation of PVAT significantly influences the rate of atherosclerotic progression. As mentioned earlier, preclinical evidence suggests that PVAT in a brown-like state under healthy conditions can protect endothelial function and mitigate plaque pathology (34, 55, 161). However, in the pathological context of obesity, PVAT loses its thermogenic protective effect and undergoes whitening, transforming into a highly pro-inflammatory pathological microenvironment (56, 59). Through paracrine pathways, it releases bioactive substances such as chemokines, directly inducing pathological remodelling of VSMCs, promoting macrophage infiltration and foam cell transformation and becoming a key driver of AS (7, 147, 162).

It is important to note that excessive or inappropriate activation of BAT may also pose potential pathogenic risks. In certain cold-acclimatised or severe metabolic stress models, cold-induced potent lipolysis, if unaccompanied by effective energy expenditure via UCP1, leads to excessive release of small, dense low-density lipoproteins into the plasma. This, in turn, plaques, triggers massive infiltration of pro-inflammatory immune cells and microvascular neogenesis within dramatically accelerating the instability of atherosclerotic plaques (122). Furthermore, variations in environmental temperature have revealed temperature-sensitive regulation of monocyte release; thermoneutral conditions reduce vascular plaque recruitment by decreasing monocyte release from the bone marrow, an immunomodulatory effect that may operate independently of the classical UCP1 systemic metabolic protection network (124). Collectively, these mechanisms indicate that targeting adipose tissue through thermogenic activation and localized PVAT inflammation resolution constitutes a cutting-edge strategy for AS intervention. Nevertheless, this therapeutic paradigm demands precise modulation of the dynamic immunometabolic equilibrium.

### 5.2 Immune responses of BAT in hypertension

At the clinical epidemiological level, the presence of active BAT in the human body shows a significant and independent negative correlation with the incidence of hypertension, accompanied by systemic improvements in systemic glucose and lipid metabolism (79). In local blood pressure regulation, pathological alterations in the PVAT secretory profile constitute the core biological link connecting obesity to the development of hypertension (54). When metabolic dysfunction occurs, PVAT loses its ability to release key anti-contractile factors such as hydrogen sulphide, instead releasing large amounts of adipocyte-derived contractile factors, which directly lead to abnormally elevated microvascular tension (162, 163) (**Figure 3B**).

The local renin-angiotensin-aldosterone system(RAAS) plays a central pathogenic role in the immunometabolic interactions of PVAT. Active local RAAS networks are present in both white and brown-like PVAT; systemic or local angiotensinogen overexpression can directly induce hypertension, adipocyte hypertrophy and inflammation, whilst targeted inhibition of the RAS pathway can effectively reverse this pathological phenotype (164). As a potent pro inflammatory and oxidative stress inducer, angiotensin II significantly amplifies obesity related disruptions in the perivascular microenvironment (54). When PVAT loses its BAT characteristics, paracrine crosstalk between adipocytes and VSMCs undergoes severe alterations, significantly enhancing the vasoconstrictive response to angiotensin II and leading to treatment-resistant hypertension that is entirely independent of changes in body weight (165). Under angiotensin II-mediated stress, SIRT3 deficiency in bone marrow-derived macrophages within PVAT further activates the NLRP3 inflammasome, exacerbating local inflammatory infiltration and perivascular fibrosis (71).

The development of hypertension is accompanied by endothelial dysfunction, severe oxidative stress and disordered infiltration of immune cells. Arterial hypertension drives a phenotypic reversal in PVAT, characterised by a complete loss of anti-contractile function, accompanied by increased hydrogen peroxide production, reduced nitric oxide bioavailability and abnormal upregulation of NOX-1 expression (166). With progressive ageing and rising blood pressure, large-scale immune cell infiltration occurs within the PVAT microenvironment, including a significant increase in the total number of leukocytes, pathogenic macrophages and natural killer cells, directly linking local inflammation to vascular resistance (167). In response to this pathological process, the A1 scavenger receptor-dependent signalling pathway can directly curb the phenotype of obesity-related hypertension by intervening in perivascular macrophages to inhibit the excessive production of vascular endothelial growth factor (168).

Targeting the remodelling of BAT and PVAT offers highly promising intervention strategies for the treatment of hypertension and vascular endothelial damage. Restoring a healthy PVAT microenvironment can re-enable its capacity to secrete vasodilatory factors; aerobic exercise can significantly improve PVAT dysfunction caused by a high-fat diet and restore the vasodilatory response of the vascular wall to acetylcholine (169). Concurrently, enhancing leptin-signal-mediated thermogenesis in BAT can significantly lower systolic blood pressure, slow the progression of AS and restore endothelium-dependent vasodilation (151). In terms of pharmacological interventions, lactoferrin supplementation can inhibit PVAT inflammatory pathways, reduce levels of vascular endothelial adhesion molecules and lower systolic blood pressure (170); the gut microbial metabolite butyrate, by reshaping and activating BAT endocrine function, produces secretory factors with antihypertensive effects to alleviate cold-induced hypertension (171). Furthermore, the BAT-derived metabolite quinolinic acid has been shown to specifically modulate renal haemodynamics and blood pressure responses in hypertensive models (172). Conversely, if BAT function declines, not only is it unable to buffer blood pressure increases, but it also significantly accelerates pathological cardiac remodelling in the context of hypertension (1).

### 5.3 Immune responses of BAT in vasculitis

PVAT serving as a crucial functional platform enveloping the vascular wall, exhibits pathological remodelling closely associated with the development of vasculitis and aortic aneurysms. High-density PVAT accumulation is frequently observed around abdominal aortic aneurysms and the shift in its transcriptomic signature towards the pathological characteristics of thoracic aortic aneurysms constitutes a significant independent risk factor driving aneurysm formation and vascular wall structural disruption (75). During this pathological process, PVAT not only loses its original vasoprotective function but also transforms into a pro-inflammatory microenvironment, driving the infiltration of local immune cells. Studies have demonstrated that transplantation of periaortic adipose tissue from the thoracic aorta can inhibit VSMCs apoptosis and effectively delay the pathological progression of aortic aneurysms by releasing autocrine components from specific BAs (123). Furthermore, the ‘beige-like’ remodelling spontaneously induced in PVAT under immunological stress exerts a barrier function, protecting the vascular wall from pathological fibrosis and inflammatory remodelling by fine-tuning local pro-inflammatory and anti-inflammatory cascades (58) (**Figure 3C**).

### 5.4 Immune responses of BAT in hypertrophic cardiomyopathy and heart failure

Compensatory failure of BAT caused by chronic heart failure exacerbates cardiac deterioration; therefore, targeting the restoration of BAT function has emerged as a potential therapeutic strategy (139, 165). As mentioned earlier, 12,13-diHOME and FGF21, which are specifically released by BAT, are key mediators in maintaining myocardial homeostasis. In patients with heart failure, reduced circulating levels of 12,13-diHOME often portend a poorer clinical prognosis, highlighting the critical role of the loss of endocrine protective function in accelerating cardiac pathological remodelling (2, 126). FGF21, as another key batokine, effectively mitigates cardiac fibrotic remodelling through its anti-hypertrophic and cardioprotective effects, demonstrating clear efficacy particularly in models of ischaemic cardiac injury (2, 121). Furthermore, adipose-derived stem cell patches promote gap junction remodelling by precisely regulating the polarisation transition of macrophages resident in the infarct zone, thereby preventing the progression of heart failure at both the histological and electrophysiological levels (155).

Conversely, the loss of BAT’s endocrine protective function directly accelerates cardiac pathological remodelling. Under angiotensin II-mediated haemodynamic stress, mice with brown fat-specific ADRB3 knockout exhibited significantly exacerbated myocardial hypertrophy and fibrosis and this cardioprotective effect could be partially attenuated by inhibiting the abnormal loading and delivery of iNOS in exosomes (142). Lipotoxicity is also a major contributor to myocardial dysfunction; free fatty acids released from excessive epicardial fat accumulation can be taken up by cardiomyocytes, leading to ectopic lipid accumulation, which in turn triggers cardiomyocyte apoptosis and disorganisation (173) (**Figure 3D**). Metabolic inflammation-driven activation of the NLRP3 inflammasome is also a key mechanism of myocardial injury. Mice lacking UCP1 exhibit exacerbated ventricular remodelling; transplantation of functional BAT significantly improves the survival and repair of damaged myocardium, confirming the anti-inflammatory and metabolic homeostasis protective value of BAT for the heart (117).

## 6 Pharmacological interventions targeting the BAT-immune axis and their potential for clinical translation

### 6.1 Immunological benefits and limitations of conventional BAT activators

Beyond the primary pharmacological agents, several foundational mechanisms and emerging therapeutic dimensions warrant distinct attention. However, evaluating the clinical feasibility of these systemic pharmacological interventions (**Table 3**) hinges heavily on a critical assessment of their advantage-to-limitation ratio. Although classic adrenergic agonists drive rodent BAT activation, the translational relevance of β3-AR signaling in adult humans remains highly controversial. Transcriptomic profiling reveals that human BAT expresses significantly lower β3-AR levels than murine tissues, with β2-adrenergic receptors instead emerging as the predominant thermogenic mediators (12, 18). Consequently, numerous rodent-derived β3-agonists exhibit clinical inefficacy. Furthermore, while the β3-agonist mirabegron stimulates human BAT, the requisite supratherapeutic doses inevitably provoke off-target cardiovascular adversities, notably tachycardia and elevated systolic blood pressure, secondary to β1-receptor cross-reactivity (176, 195). Therefore, systemic β3-AR activation proves unviable as a standalone therapy, dictating a critical reassessment of adrenergic targets for cardiovascular patients. .

**Table 3:** Pharmacological agents and therapeutic strategies targeting adipose tissue thermogenesis and browning.

Drug	Target/pathway	Evidence source	Research stage	Core efficacy	Limitations	Refs
CL316,243	β3-AR/Lace1	Mouse	Experimental	Induces WAT browning to ameliorate lipid metabolism and hypercholesterolemia. Synergizes with PCSK9 inhibitors to optimize lipid profiles and attenuate AS. Lace1 deletion facilitates lactate utilization to drive browning	Potential cardiovascular side effects	(140, 158, 174)
Mirabegron	β3-AR/Pcpe2	Human and mouse	Clinical	Activates BAT to improve cardiovascular function and mitigate AS risk. Upregulates HDL-C uptake via the Pcpe2/SR-BI pathway	Requires supratherapeutic doses for efficacy, provoking cardiovascular side effects	(156, 175, 176)
Mirabegron + Bisoprolol	β3-AR + β1-AR antagonism	Human	Clinical	Bisoprolol effectively counteracts mirabegron-induced cardiovascular side effects while maintaining or potentiating BAT stimulation	Not specified	(176)
Mirabegron + Metformin	β3-AR + AMPK	Mouse	Experimental	Synergistically ameliorates diet-induced obesity. Suppresses energy intake via sympathetic-immune interactions and enhances BAT-mediated energy expenditure	Not specified	(90, 177)
Tesaglitazar	Dual PPARα/γ	Human and mouse	Experimental	Synergistically activates PPARα and PPARγ to robustly induce WAT browning and reverse hepatic steatosis *in vivo*	Potential toxicity	(178)
Resmetirom	THR-β	Human	Clinical	Specifically targets hepatic THR-β to alleviate lipid overload and steatosis. Approved for NASH treatment	Long-term safety requires evaluation	(17)
BAT Activators + Colesevelam	Thermogenesis + Bile acid binding	Mouse	Experimental	Co-administration promotes bile acid reabsorption, reversing BAT activation-induced hypercholesterolemia and consequently retarding AS	Potential gastrointestinal side effects	(179)
Metformin	AMPK	Human and mouse	Experimental	Modulates the endocrine activity of PVAT. Suppresses inflammation, increases oxygen consumption and improves vascular function	High interindividual variability	(180)
Simvastatin NPs	Macrophage immunomodulation/Local delivery	Mouse	Experimental	Employs polymeric nanoparticles for local delivery to specifically target inflammatory macrophages, thereby mitigating inflammation and inducing adipose browning	Challenges in clinical translation	(181)
Telmisartan	Bcat2 inhibition	Mouse	Experimental	Directly binds and inhibits Bcat2 activity to reduce PRDM16 deacetylation, robustly promoting WAT browning and alleviating obesity	Not specified	(164, 182)
SGLT2i/GLP-1 RAs	SGLT2/GLP-1 receptor	Human	Clinical	Remodels and visceral fat to induce an anti-inflammatory phenotype and browning, exerting cardioprotective effects	Potential side effects	(36, 183)
Senolytics(DQ)/Paquinimod	Senescent cell clearance/S100A8 inhibition	Mouse	Experimental	Eliminates senescent immune cells or inhibits S100A8 secretion to remodel the BAT sympathetic axis, reversing obesity, insulin resistance and cardiovascular dysfunction	Long-term safety requires evaluation	(105, 184)
N-acyl amino acids	Mitochondrial uncoupling/ANT carrier	Human and mouse	Experimental	Acts as an effective mitochondrial uncoupler in murine models. Triggers adverse bioenergetic side effects in human adipocytes	Species differences exist	(30)
Guanosine	KEAP1-NRF2 pathway/Ferroptosis	Mouse	Experimental	Prevents KEAP1-mediated NRF2 degradation to bolster BAT antioxidant capacity, resisting adipose tissue ferroptosis and enhancing thermogenesis	Not specified	(185)
Inosine	cAMP-PKA signaling/Purine metabolism	Human and mouse	Experimental	Released by apoptotic BAs to stimulate energy expenditure in healthy BAs via the cAMP-PKA pathway and induce WAT browning	Potential off-target effects	(152)
Polyamines	Macrophage metabolism/eIF5A modification	Human and mouse	Predictive	Regulates macrophage pleiotropy and oxidative metabolism in BAT. Supports adaptive thermogenesis and immunometabolic homeostasis under cold stimulation	Not specified	(186)
D-Mannose	ACSS2-PPARγ-SIRT1-UCP1 axis	Human and mouse	Experimental	Activates ACSS2 and couples PPARγ activation with its degradation to maintain PPARγ activity homeostasis, inducing robust WAT beiging and BAT thermogenesis	Not specified	(187)
Adipose Tissue-Targeted Delivery	Nanoparticles/Microneedle technology	Human and mouse	Predictive	Utilizes advanced delivery systems to directly localize pro-browning agents to adipose tissue, circumventing systemic cardiovascular side effects	Challenges in clinical translation	(188)
Corylin	SIRT1 activation/β3-AR pathway	Mouse	Experimental	A natural flavonoid that promotes WAT browning via SIRT1 and β3-AR pathways, alleviating obesity and hyperlipidemia while reversing insulin resistance	Low bioavailability	(33)
Gegen Huazhuo Tang	TGR5 activation/7-KDCA inhibition	Mouse	Experimental	A traditional Chinese medicine formula that promotes WAT browning by inhibiting the gut microbiota metabolite 7-KDCA and upregulating TGR5 expression	Complex composition	(189)
Stachyose + Theaflavins	Gut microbiota modulation/UCP1 thermogenesis	Mouse	Experimental	Stachyose enhances the bioavailability of theaflavins to synergistically ameliorate high-fat diet-induced obesity and glucolipid metabolic dysregulation, concurrently upregulating UCP1	Not specified	(190)
Dichloroacetate (DCA)	PDK inhibition/AMPK activation/Hepatic FGF21	Mouse	Experimental	Acutely activates AMPK to promote hepatic FGF21 expression. Upregulates Dio2/Ucp1 to trigger BAT activation, mitigating obesity and retarding AS	Potential neurotoxicity	(118)
XMU-MP-1	STK3/STK4 inhibition/Mitophagy	Human and mouse	Experimental	Inhibits STK3/STK4 kinases to remove mitochondrial constraints. Increases mitochondrial mass, modulates mitophagy and stabilizes UCP1 in beige adipose tissue	Potential systemic side effects	(191)
Dotinurad	URAT1 inhibition/UCP1	Mouse	Experimental	A urate-lowering drug that significantly alleviates hepatic steatosis, prevents BAT whitening and improves insulin resistance	Long-term safety requires evaluation	(192)
BATSP1	Adipocyte targeting/Secreted peptide	Human and mouse	Experimental	A metabolic peptide secreted by BAT that promotes BAT thermogenesis and WAT browning in a cell-autonomous manner, preventing diet-induced obesity	Restricted administration routes	(21).
Hyperoside	Dlat-AMPK-PGC1α axis	Human and mouse	Experimental	A natural phytochemical that directly binds Dlat protein, triggering adaptive thermogenesis in adipose tissue via the Dlat-AMPK signaling axis to curb obesity	Potential off-target effects	(193)
β-carotene	β3-AR/p38 MAPK/SIRT1 axis	Mouse	Experimental	Significantly upregulates core brown/beige adipocyte markers UCP1and PRDM16 *in vitro*. Robustly promotes WAT beiging by enhancing thermogenesis	Not specified	(194)

This table summarizes current experimental and clinical interventions modulating BAT activity and WAT browning.

7-KDCA, 7-ketodeoxycholic acid; ACSS2, acyl-CoA synthetase short-chain family member 2; AMPK, AMP-activated protein kinase; ANT, adenine nucleotide translocator; β3-AR, β3-adrenergic receptor; BAT, brown adipose tissue; Bcat2, branched-chain amino acid transaminase 2; cAMP, cyclic adenosine monophosphate; Dlat, dihydrolipoamide S-acetyltransferase; EAT, epicardial adipose tissue; eIF5A, eukaryotic translation initiation factor 5A; ERα, estrogen receptor alpha; FGF21, fibroblast growth factor 21; GLP-1 RAs, glucagon-like peptide-1 receptor agonists; HDL-C, high-density lipoprotein cholesterol; KEAP1, kelch-like ECH-associated protein 1; NASH, non-alcoholic steatohepatitis; NPs, nanoparticles; NRF2, nuclear factor erythroid 2-related factor 2; p38 MAPK, p38 mitogen-activated protein kinase; Pcpe2, procollagen C-endopeptidase enhancer 2; PDK, pyruvate dehydrogenase kinase; PGC1α, peroxisome proliferator-activated receptor gamma coactivator 1-alpha; PKA, protein kinase A; PPAR, peroxisome proliferator-activated receptor; PRDM16, PR domain containing 16; PVAT, perivascular adipose tissue; SGLT2i, sodium-glucose cotransporter-2 inhibitors; SIRT1, sirtuin 1; SR-BI, scavenger receptor class B type I; STK, sterile 20-like kinase; TGR5, Takeda G-protein-coupled receptor 5; THR-β, thyroid hormone receptor beta; UCP1, uncoupling protein 1; URAT1, urate transporter 1; WAT, white adipose tissue.

To maximize therapeutic efficacy while mitigating adverse effects, combination therapies are actively explored. For instance, co-administering mirabegron with the β1-antagonist bisoprolol reduces cardiovascular toxicities but inadvertently blunts mirabegron-stimulated BAT oxidative metabolism, underscoring the complexity of clinical optimization (176). Similarly, pairing BAT activators with bile acid sequestrants such as colesevelam counteracts activation-induced hypercholesterolemia, yielding a balanced therapeutic response despite potential gastrointestinal adversities (179). In contrast, repurposing clinically approved metabolic modulators, including SGLT2 inhibitors, GLP-1 receptor agonists, and THR-β agonists, currently offers the highest translational feasibility. By bypassing initial safety hurdles, these agents confer profound cardioprotective and anti-inflammatory benefits via visceral and epicardial adipose tissue remodeling. Consequently, their well-documented tolerability and broad efficacy demonstrate substantial clinical viability (17, 36, 183).

Concurrently, diverse metabolic modulators facilitate critical cellular phenotypic shifts that actively oppose inflammation and ferroptosis within the local adipose microenvironment (34). In the realm of natural pharmacognosy, specific plant derivatives demonstrate substantial metabolic efficacy. Anthocyanin oligomers effectively induce adipose thermogenesis (196), while resveratrol upregulates SIRT1 levels to enhance tissue energy expenditure *in vitro* and in rodents, thereby elevating local thermogenic markers and curbing focal lipid accumulation (6).

Crucially, brown precursor adipocytes isolated from elderly and obese demographics retain a robust *in vitro* capacity for UCP1 upregulation. This biological resilience indicates that expanding and stimulating residual precursor cell populations using classical endocrine therapeutics, novel small molecules, or innovative strategies targeting PVAT represents a highly promising frontier for reversing AS and advanced cardiometabolic diseases (7, 197).

### 6.2 Nanomaterial and liposome-mediated delivery systems targeting BAT

The scientific community has not only identified a large number of novel brown fat activators, but is also actively developing nanotechnology-based targeted drug delivery systems. These aim to circumvent the cardiovascular and immunological off-target side effects associated with traditional systemic administration, whilst significantly enhancing the efficacy of the drugs in the local adipose tissue (23). To achieve highly specific targeting, researchers screened a phage display library to identify the specific adipose-homing peptide CKGGRAKDC, which can precisely recognise and bind to the PHB receptor on the surface of vascular endothelial cells in WAT, thereby laying the foundation for ligand-targeted remodelling of the cardiovascular metabolic microenvironment (188). Based on this, a dual-targeted nanosystem (Rs-NPs) loaded with rosiglitazone was constructed, with the fat-homing peptide and the cell-penetrating R8 peptide simultaneously modified on its surface. This system successfully and potently drove the specific browning and remodelling of adipose tissue *in vivo* and *in vitro*, whilst avoiding off-target toxicities such as systemic oedema and bone loss (188). Mechanisms mediated by nucleic acid aptamers and peptides endow specific nanocarriers with exceptionally high targeting efficacy. Targeting adipose-specific populations of mesenchymal stem cells, specific peptides that recognise the ADCN receptor were utilised to mediate the precise endocytosis of resveratrol-containing nanoparticles, thereby promoting the *de novo* differentiation of progenitor cells into active beige adipocytes (188). The Adipo-8 aptamer, which binds with high affinity to mature adipocytes, was conjugated to a PEG-PLGA carrier, significantly increasing the effective accumulation of the drug within target cells and reducing systemic dissipation (188). For the plant monomer tripterine, which is highly insoluble in water and has poor gastrointestinal stability, a triple-layered oral nanodelivery system not only protects the drug molecule from degradation but also facilitates its absorption through the intestine to target the adipose vascular network directly (188). Furthermore, a therapy utilising liposomes to precisely deliver triiodothyronine to adipose tissue can completely circumvent the reflexive inhibition of sympathetic nerve impulses caused by systemic administration, thereby safely and potently activating the browning network without cardiovascular toxicity in murine models (153) (154). Emerging *in vitro* three-dimensional adiposphere culture technology, due to its ability to highly replicate the advanced phenotypic characteristics of real cells, provides a translational model superior to traditional two-dimensional monolayer cells for in-depth validation of these cutting-edge nano-targeted delivery effects (37).

Topical delivery systems based on sustained-release polymer microspheres and hydrogel matrices demonstrate exceptional engineering advantages in achieving long-lasting regional immune modulation and metabolic improvement. To avoid the cardiovascular side effects induced by traditional systemic administration, polylactic-co-glycolic acid (PLGA) microspheres loaded with the highly selective β3 receptor agonist mirabelton are capable of maintaining stable release at the injection site for up to several days (195). Targeted injection of these microspheres successfully induced intense browning and specifically upregulated the expression of the thermogenic marker UCP1, whilst significantly reducing systemic drug exposure in plasma, thereby successfully avoiding adverse cardiovascular risks such as elevated systolic blood pressure and increased heart rate caused by drug diffusion (195). Similar polymeric nanoparticles loaded with simvastatin demonstrated the ability to maintain long term structural stability and controlled release in serum for several weeks; by slowly releasing the drug into the local matrix, they reversed the phenotypic polarisation of macrophages in the microenvironment from M1 to M2, significantly reducing the secretion of pro-inflammatory factors (181). This immunopharmacological delivery system directly regulates immune activity, inducing fat browning and reducing body weight whilst effectively inhibiting the progression of obesity-related inflammation and limiting abnormal lipid synthesis, demonstrating immense translational potential (181). Furthermore, utilising tissue nano-transfection technology to continuously deliver and upregulate specific lipid mediators 12,13-diHOME (126), or utilising a novel hydrogel material to precisely deliver and encapsulate FSTL1 protein patches within the local microenvironment surrounding the abdominal aorta (120), can non-invasively and with extreme precision limit pathological dilation of the vascular wall at the target site and physically block aneurysm progression through microecological intervention mechanisms, demonstrating a novel drug delivery paradigm for combating cardiovascular remodelling. Targeted nucleic acid-modified and recombinant EVs represent cutting-edge clinical tools for achieving gene-level reversal of adipose tissue and cell-free immunological intervention. The local delivery of modified sequences using microRNA-targeted delivery strategies or lipid nanocarriers for prospective applications provides a broad avenue for gene transformation to precisely activate browning of adipose tissue and intervene in cardiovascular and metabolic diseases (198, 199). The use of adeno-associated viruses to specifically deliver vectors containing miR-9-3p sponge sequences to adipose tissue enables the precise adsorption of pathogenic nucleic acids, successfully rescuing cross-organ synaptic loss and cognitive impairment caused by abnormal adipose remodelling (83). *In situ* injection of adeno-associated viruses to specifically knockdown Notch1 in iWAT or to target Aldh1a1 in mature adipocytes significantly increases UCP1 levels in response to cold stimulation and substantially improves systemic body weight and dysregulated glucose and lipid metabolism (43, 200). Similarly, endothelial cell-specific delivery of DEL-1 (201) or viral vector-mediated targeted overexpression of Foxj3 in adipocytes (92) can effectively restore the thermogenic response and significantly increase whole-body energy expenditure, thereby enhancing the body’s metabolic resilience under a high-fat diet challenge. In the field of gene editing, non-mutation-dependent lead editing and multi-activation complex systems have overcome the limitations of traditional gene knockout, which is prone to inducing irreversible off-target effects. The CRISPRa system, delivered via adeno-associated virus, possesses the cutting-edge capability to precisely modify and upregulate Fgf21 and Fndc5 *in vivo (*153). Combining CRISPRa and siRNA co-delivery technology, along with CRISPR mediated precise base editing of DNA, has enabled bidirectional precise regulation of key gene expression in adipocytes (8). Human brown-like adipocytes, engineered through *in vitro* modification, are even capable of systematically preventing and correcting diet-induced obesity syndrome and cardiovascular complications upon implantation into a host (8). On the other hand, small EVs extracted from adipose tissue and specifically engineered are emerging as a novel nanoscale natural delivery vehicle, playing an irreplaceable role in the targeted delivery of therapeutic molecules (134). Exogenous intravenous infusion of small EVs loaded with the NPM3 signalling protein demonstrates the translational potential of cell-free therapy to improve defects in adipose browning and for direct application in obesity treatment (136),whilst small vesicles loaded with a plasmid encoding the specific kinase dominant-negative mutant AMPKα2-DN are capable of precisely modifying and blocking target molecular channels, significantly reducing the infarct size in ischaemic models and promoting the recovery of neural function (202).

### 6.3 The limitations of BAT-based therapies in humans

Despite the profound cardiovascular and immunometabolic benefits of targeting the BAT immune axis in rodents, clinical translation to adult humans warrants critical caution. Unlike murine models, 18F-fluorodeoxyglucose PET/CT reveals human interscapular BAT peaks during infancy (203) and undergoes progressive involution throughout early adulthood (45), severely limiting its active mass and distribution. Furthermore, aging exacerbates these anatomical constraints by diminishing overall BAT function and mitochondrial capacity (70). Crucially, obese populations representing the primary therapeutic demographic exhibit severe pathological adipose remodeling. Obesity-associated BAT whitening precipitates mitochondrial dysfunction, reduced energy expenditure, and ectopic lipid deposition, establishing a chronic pro-inflammatory state that amplifies cardiovascular risks including hypertension and atherosclerosis (34, 72). Given these physiological barriers and the inverse correlation between active BAT and metabolic disorders, activating this minimal, dysfunctional depot alone remains insufficient against advanced cardiometabolic diseases (15). Therefore, overcoming these inherent constraints is an imperative prerequisite for future clinical translation.

## 7 Discussion

Redefining BAT from a sheer thermogenic effector to a central orchestrator of cardiovascular immunity fundamentally transforms cardiometabolic interventions. Although classical UCP1-mediated energy expenditure retains therapeutic relevance, secretome-driven cross-organ immunomodulation presents a more sophisticated and potent target. However, applying BAT biology to cardiovascular therapeutics encounters substantial difficulties. Translating rodent-derived BAT mechanisms to adult humans remains controversial, given the sparse, heterogeneous distribution of active human BAT and its profound decline with age and obesity. Furthermore, whether activating this limited, functionally impaired reserve confers weight-independent cardiovascular protection remains debated. While systemic β-adrenergic agonists exhibit robust metabolic efficacy, they inevitably precipitate severe cardiovascular toxicities. Consequently, clinical paradigms must decisively transition from blunt systemic sympathetic stimulation toward localized microenvironmental remodeling.

A critical frontier involves decoding the spatiotemporal dynamics of batokine release across distinct cardiovascular pathological stages. Additionally, the precise molecular and epigenetic triggers driving BAT whitening and its transition from a cardioprotective organ to a pro-inflammatory depot during atherosclerosis and heart failure require elucidation. Because the bidirectional heart-BAT axis indicates that the cardiovascular system actively influences adipose plasticity under stress, preserving BAT function before heart failure-induced metabolic exhaustion represents an underexploited therapeutic window. Moreover, the profound heterogeneity of tissue-resident macrophages within discrete adipose depots demands rigorous interrogation via spatial transcriptomics and single-cell multi-omics. Deciphering how specific neuro-immune reflex arcs sustain sympathetic innervation may therefore yield unprecedented strategies to reverse aging-associated adipose whitening and neurodegeneration.

Overcoming these translational barriers mandates the rigorous refinement of adipose-targeted delivery platforms. Advanced nanotechnologies, including polymeric nanoparticles, aptamer-conjugated liposomes, and engineered small EVs, exhibit immense potential to confine browning agents within specific adipose beds. Integrating these delivery vehicles with CRISPR-based epigenetic editing offers a revolutionary cell-free intervention paradigm. Future research must prioritize validating these advanced interventions in large animal models or human adipose organoids to accurately capture physiological complexities. Ultimately, shifting the therapeutic objective from increasing energy expenditure to precisely modulating the BAT-cardiovascular immune axis will deepen our understanding of cardiometabolic and immune microenvironments.
